# Inhibition of SMYD2 suppresses tumor progression by down-regulating microRNA-125b and attenuates multi-drug resistance in renal cell carcinoma

**DOI:** 10.7150/thno.37628

**Published:** 2019-10-22

**Authors:** Libin Yan, Beichen Ding, Haoran Liu, Yangjun Zhang, Jin Zeng, Junhui Hu, Weimin Yao, Gan Yu, Ruihua An, Zhiqiang Chen, Zhangqun Ye, Jinchun Xing, Kefeng Xiao, Lily Wu, Hua Xu

**Affiliations:** 1 Department of Urology, Tongji Hospital, Tongji Medical College, Huazhong University of Science and Technology, Wuhan, China;; 2 Institute of Urology of Hubei Province, Wuhan, China; 3 Department of Urinary Surgery, First Affiliated Hospital of Harbin Medical University, Harbin, Heilongjiang, China.; 4 Department of Urology, The First Affiliated Hospital of Xiamen University, Xiamen, China.; 5 Department of Urology, The People's Hospital of Shenzhen City, Shenzhen, China.; 6 Department of Molecular and Medical Pharmacology, David Geffen School of Medicine, University of California at Los Angeles, Los Angeles, USA

**Keywords:** SMYD2, Renal cell carcinoma, MicroRNA, Multidrug resistance

## Abstract

SMYD2 is a histone methyltransferase that has been reported to be an important epigenetic regulator. This study aims to investigate SMYD2 as a prognostic indicator of clear cell renal cell carcinoma (ccRCC) and explore its role in tumorigenesis and multi-drug resistance.

**Methods**: Tumor specimens, clinicopathologic information, and prognostic outcomes of 186 ccRCC patients from three hospitals in China were collected for SMYD2 immunohistochemistry staining, Kaplan-Meier analysis, and Cox proportional hazards-regression analysis. MicroRNA (miRNA)-microarray profiling identified differentially expressed miRNAs in renal cancer cells subjected to SMYD2 knockdown or treatment with the SMYD2 inhibitor AZ505. The effects of SMYD2 and candidate SMYD2-mediated miRNAs on renal cancer cell proliferation, migration, clonogenicity, and tumorigenicity were determined via cell-function assays and murine xenograft experiments. The half-inhibitory concentrations (IC_50_) of five antineoplastic drugs (cisplatin, doxorubicin, fluorouracil, docetaxel, and sunitinib) in AZ505-treated and control cells were calculated, and the effects of SMYD2 inhibition on P-glycoprotein (P-gP) expression and multiple-drug resistance were verified.

**Results**: SMYD2 was overexpressed and acted as an oncogene in ccRCC. High SMYD2 expression correlated with a high TNM stage (P = 0.007) and early tumor relapse (P = 0.032). SMYD2 independently predicted a worse overall survival (P = 0.022) and disease-free survival (P = 0.048). AZ505 inhibited the binding of SMYD2 to the miR-125b promoter region (based on chromatin immunoprecipitation assays) and suppressed ccRCC cell migration and invasion by inhibiting the SMYD2/miR-125b/DKK3 pathway. SMYD2 and miR-125b inhibition acted synergistically with anticancer drugs via P-gP suppression in vitro and in vivo.

**Conclusions**: These findings suggested that SMYD2 plays an important role in ccRCC development and could be a potential biomarker for the treatment and prognosis of RCC.

## Introduction

Renal cell carcinoma (RCC) remains the seventh most common form of cancer, causing more than 140,000 deaths per year 1, 2. Clear cell renal cell carcinoma (ccRCC), a renal cancer subtype, accounts for 75-80% of all RCCs and is mainly characterized as an organ-confined disease. Surgical removal of local ccRCC tissues typically results in long-term disease-free survival (DFS); however, the survival rates of patients with advanced ccRCC are often poor because of metastasis or recurrence resulting from resistance to traditional chemotherapy and radiotherapy after primary radical surgery 3-5. Therefore, it is important to understand the potential molecular mechanisms underlying malignant ccRCC and determine new effective therapeutic strategies.

Multidrug resistance (MDR) is a significant obstacle to providing effective chemotherapy to RCC patients. RCC ranks among the most chemo-refractory cancers, and the expression of multidrug resistance-1(MDR-1) has been shown to be abnormally upregulated in a high percentage of RCC patients 6. MDR largely limits the effectiveness and application of a broad range of conventional drugs 7. Classic MDR is associated with the overexpression of P-glycoprotein (P-gP), resulting in an increased efflux of chemotherapeutic agents from cancer cells. Inhibiting P-gP as a method to reverse MDR in cancer patients has been studied extensively, but the results in RCC patients have been disappointing thus far^8^.

Histone methyltransferases (HMTs) represent a subclass of epigenetic regulators that control histone lysine methylation 9, 10. SET and MYND domain-containing protein 2 (SMYD2) is a member of the SMYD-methyltransferase family of proteins and methylates the histone on lysine 36 and 4 (H3K36 and H3K4) 11. SMYD2 can also methylate non-histone proteins, such as the tumor-suppresser proteins P53, RB1, and PTEN, and can activate the oncoprotein PARP1 at K370, K860, K313, and K528^12^-^15^. SMYD2 has been shown to act as an oncogene in many cancers, including esophageal squamous cell carcinoma (ESCC), bladder cancer, gastric cancer, triple-negative breast cancer, and hepatocellular carcinoma 16-19. It has also been reported that SMYD2 affects the resistance to platinum-based chemotherapy in patients with advanced-stage non-small cell lung cancer 20, and that SMYD2 inhibition enhances the efficacy of doxorubicin against ESCC *in vitro* 21. AZ505 is a specific SMYD2 inhibitor identified by high-throughput chemical screening, which binds to the peptide-binding groove of SMYD2, competing with the peptide substrate 22.

MicroRNAs (miRNAs) are small non-coding RNAs that act as endogenous silencers of various target genes 23. Intriguingly, miRNA expression can be epigenetically regulated, and epigenetically modified protein-coding genes can also be targeted by miRNAs, suggesting an interconnected regulatory mechanism between epigenetics and miRNAs 24. However, the miRNA-expression profiles altered by SMYD2 inhibition remain unknown.

Till date, little is known about the role of SMYD2 in ccRCC. In this study, we investigated the potential of SMYD2 to serve as a prognostic marker for ccRCC by determining the SMYD2 expression in ccRCC samples and correlating its expression with various clinicopathologic factors. Additionally, to investigate the molecular mechanisms underlying the anticancer effects of SMYD2 inhibitors, miRNA-expression profiles in ccRCC cells were analyzed after their treatment with AZ505. Our findings are therapeutically relevant and have implications for future clinical practices with regards to ccRCC treatment.

## Materials and Methods

### Patients and tissue samples

Six pairs of ccRCC tissues and matched adjacent normal tissues were obtained from ccRCC patients treated at the Department of Urology at the Tongji Hospital (Wuhan, China) after they provided written informed consent. All samples were stored in liquid nitrogen before protein extraction. Paraffin-embedded tumor specimens and the follow-up information of a total of 186 patients were obtained following the retrieval of their pathology reports (Table S1). All patients were diagnosed with ccRCC and underwent curative surgery at the Wuhan Tongji Hospital (Wuhan, China), Chinese PLA General Hospital (Beijing, China), or the First Affiliated Hospital of the Harbin Medical University (Harbin, China). This study was registered in the Chinese Clinical Trial Registry (No. ChiCTR1800017391, http://www.chictr.org.cn/), and was carried out in accordance with the ethical standards of the Helsinki Declaration, and approved by the Tongji Hospital of the Huazhong University of Science and Technology (Wuhan, China) Ethics Review Committee (reference TJ-IRB20180619). Written informed consent was obtained from each patient prior to conducting all the study-specific investigations.

### Immunohistochemistry (IHC) and histopathological assessments

IHC was uniformly performed to evaluate the SMYD2 protein expression in the ccRCC tumor samples using the Bond Polymer Refine Detection System (Leica Biosystems, Wetzlar, Germany). Samples incubated in phosphate-buffered saline (PBS) lacking the primary antibody served as the negative controls in this detection system. Three independent pathologists blinded to the clinicopathological information performed the IHC analysis for SMYD2, and the top three areas with the strongest SMYD2 expression were selected to assess each tissue sample because of the heterogeneity of ccRCC. The immune-reactive score (IRS), which represents the intensity and quantity of the stained cells, was implemented to quantify the staining level of SMYD2, as previously reported 25. Samples with an IRS score of ≤ 3 were considered as those with low SMYD2 expression and samples with an IRS score of > 3 were considered as those with high SMYD2 expression. IHC was also utilized to assess the protein expression levels of Ki67 and P-glycoprotein (P-gP). Antibodies against the following targets were used for IHC: SMYD2 (1:50, Proteintech, Rocky Hill, NJ, USA, 21290-1-AP), Ki67 (1:6000, Proteintech, 27309-1-AP), and P-gP (1:50, Abcam, Cambridge UK, ab170904).

### The Cancer Genome Atlas (TCGA) data processing, gene ontology analysis, and pathway enrichment analysis

All TCGA data used in this research, including the SMYD2 gene expression data of 71 paired ccRCC samples, miRNA expression data, and corresponding clinicopathological data of 326 ccRCC samples, were downloaded from the UCSC XENA database (http://tcga.xenahubs.net). The Limma and DEseq packages in the R software (version 3.0.0) were used for the differential miRNA expression analysis. Significantly dysregulated miRNAs were defined using a cutoff |log_2_ fold-change| value of > 1 and P value of < 0.05.

The target genes of the miRNAs were retrieved from the miRDB (http://mirdb.org), miRbase (http://www.mirbase.org), and TargetScan databases (http://www.targetscan.org). The Database for Annotation, Visualization, and Integrated Discovery (DAVID) (https://david.ncifcrf.gov) online tool was utilized to perform the Gene Ontology (GO) and Kyoto Encyclopedia of Genes and Genomes (KEGG) analysis for these miRNA target genes. Gene sets or pathways with P values < 0.05 were retained for further analysis.

### Cell culture

HK-2 and HEK293T cells were maintained in Dulbecco's modified Eagle's medium supplemented with 10% fetal bovine serum and 2 mmol/L L-glutamine in a humidified atmosphere containing 5% CO_2_ at 37°C. OSRC-2 and 786-O cells were cultured in Roswell Park Memorial Institute-1640 medium supplemented with 10% fetal bovine serum and 2 mmol/L L-glutamine.

### Antibodies and reagents

Antibodies against the following proteins were used: SMYD2 (ab195365), STAT3 (ab68153), phospho-STAT3 (Y705, ab76315), DKK3 (ab186409), and P-gP (ab170904) from Abcam; β-tubulin (10068-1-AP), CDK6 (14052-1-AP), N-cadherin (22018-1-AP), and GAPDH (60004-1-lg) from Proteintech; and E-cadherin (11A24) and vimentin (BM0135) from Boster (Wuhan, China). The following reagents were used in this research: AZ505 (HY-15226), cisplatin (HY-17394), and sunitinib (HY-10255A) from MedChemExpress (Monmouth Junction, NJ, USA); and doxorubicin (A3966), 5-fluorouracil (5-FU) (A4071), and docetaxel (A4394) from APExBIO (Boston, MA, USA).

### Small interfering RNA (siRNA) and miRNA oligonucleotides

SMYD2 expression was knocked down using a pre-validated siRNA targeting SMYD2, which was synthesized by RiboBio (Guangzhou, China), and the oligonucleotide mimics/inhibitors of 6 candidate SMYD2-mediated miRNAs (miR-18a-3p, miR-2277-5p, miR-1293, miR-93-3p, miR-5706, miR-125b) were purchased from GeneCopoeia (Rockville, MD, USA). The SMYD2 siRNA, a corresponding siRNA control, and the miRNA oligonucleotides were transfected into renal cancer cell lines with the GenMute™ siRNA transfection reagent (SignaGen, Rockville, MD, USA), according to the manufacturer's instructions. The sequences of the siRNA and miRNA oligonucleotides are shown in Table S2.

### Plasmid constructs, lentiviral transduction, and transfection

A lentiviral vector driving miR-125b expression was constructed by cloning the miR-125b sequence (5′-UCCCUGAGACCCUAACUUGUGA-3′) into the pGCMV/EGFP/miR/Blasticidin vector (GenePharma, Shanghai, China). The miR-125b sponge plasmid for stable miR-125b knockdown was constructed from the pGLV3/H1/GFP vector (GenePharma) with eight antisense repeats of the miR-125b sequence (5'-TCACAAGTTAGGGTCTCAGGGA-3'), following standard protocols. To construct the human SMYD2-expression vector, human SMYD2 complementary DNA (cDNA) was amplified with polymerase chain reaction (PCR) using SMYD2 primers (Forward, 5′-CCGGAATTCATGAGGGCCGAGGGCCTCGG-3′; Reverse, 5′-ATAAGAATGCGGCCGCTCAGTGGCTTTCAATTTCCT-3′), digested with *Eco*RI and *Not*I, and ligated into the *Eco*RI and *Not*I sites of the pCDH-CMV-MCS-EF1-GFP+Puro vector (Novagen, Madison, WI, USA). All transfections were performed using Lipofectamine 3000 (Thermo Fisher Scientific, Waltham, MA, USA), following the manufacturer's instructions.

### Quantitative reverse transcription PCR (RT-qPCR), microRNA microarray profiling, and chromatin immunoprecipitation (ChIP) assays

Total cellular RNA was extracted using TRIzol reagent (Invitrogen, Carlsbad, CA, USA) and cDNAs were synthesized using the ReverTra Ace qPCR RT Kit (Toyobo, Osaka, Japan). Real-time PCR was performed using SYBR Green Realtime PCR Master Mix (Roche, Basel, Switzerland) and the ABI ViiA7 QPCR System (Applied Biosystems).

To evaluate the differentially expressed miRNAs in AZ505-treated and siSMYD2-transfected RCC cells relative to control cells, siSMYD- (n = 4), AZ505- (n = 4), and negative control (n = 4)-treated 786-O cells were subjected to miRNA microarray analysis by Kangcheng Technology (Shanghai, China), using the miRCURY LNA Array system (Exiqon, Vedbæk, Denmark). The cutoff for defining candidate downregulated miRNAs was fold-change < 0.5 and P < 0.05.

ChIP was performed using the EZ-Chromatin Immunoprecipitation Kit (Millipore, Billerica, MA, USA, #17-371) according to the manufacturer's instructions. The DNA fragments were then extracted and analyzed by PCR using the following primer pair specific for the miR-125b promoter: Forward, 5′-TCCTTGAGAGCAACACGCAG-3′ and reverse, 5′-GTGGAGTTTGAAAGTTGGAG-3′.

### Screening of candidate SMYD2-mediated miRNAs in renal cancer cells

SMYD2 methylates histone H3 on K4, which is known to be an activating modification. Thus, candidate SMYD2-mediated miRNAs were obtained from the intersection of downregulated miRNAs in AZ505-treated cells, downregulated miRNAs in siSMYD2-transfected cells, and upregulated miRNAs in ccRCC samples from TCGA.

### *In situ* hybridization (ISH)

Renal cancer paraffin sections of six samples each showing the highest and lowest SMYD2 expression levels were sent to Servicebio Company (Wuhan, China) for hsa-miR-125b-specific ISH (Figure S1), which was performed using miRCURY LNA^TM^ 5′- and 3′-DIG-labeled hsa-miR-125b detection probes and 5′- and 3′-DIG-labeled miRCURY scrambled ISH 49C (Qiagen) probes. ISH was performed according to the manufacturer's protocol (Qiagen/Exiqon, Vedbæk, Denmark).

### Cell-viability, cell-proliferation, cell-migration, and cell-invasion assays

Cell viability was assessed at 0, 24, 48, 72, and 96 h post-treatment using the 3-(4,5-dimethylthiazol-2-yl)-5-(3-carboxymethoxyphenyl)-2-(4-sulfophenyl)-2H tetrazolium, inner salt (MTS) method (Promega, Madison, WI, USA, #0000253755) according to the manufacturer's instructions. The MTS assays were performed with six replicates. Cell proliferation was estimated using the Cell-Light EdU Apollo 568 in Vitro Kit (RiboBio, #C10310-1), according to the manufacturer's instructions. Migration and invasion assays were performed using uncoated and Matrigel-coated Transwell inserts according to the manufacturer's instructions. Wound-healing assays were also performed to evaluate the migration and invasion of the cells. After the cells were cultured for 6 h, a 100-μL pipette tip was used to draw a “+” shape in the wells of the 6-well plate. Subsequently, the wells were briefly rinsed with PBS, and 2 mL of serum-free culture medium was added to each well. Photos were taken immediately after the lines were drawn in each monolayer to observe the scratches. Changes in the scratches were imaged after 48 h. The migration and invasion of cells among different groups were judged based on variations in the widths of scratches among the different treatment groups. All experiments were performed in triplicate.

### Orthotopic xenograft model, lung metastasis model, and subcutaneous xenograft model

To generate an orthotopic xenograft model, 786-O cells were trypsinized, rinsed with PBS, and resuspended in 20 mL of Matrigel diluted with precooled PBS at a 1:1 ratio. Next, eight 4-week-old BALB/c mice were anesthetized with sterilized 1% pentobarbital sodium at a dose of 10 mL/kg via intraperitoneal injection. The right kidney of each mouse was injected with 20 µL of cells for the establishment of the orthotopic xenograft model. Eight mice were randomly assigned to an AZ505-treated group or a control group. Nude mice in the AZ505-treated group were administered with 40 mg/kg AZ505 every two days via intraperitoneal injection, while the mice in the control group were administered the same volume of dimethyl sulfoxide (DMSO). The animals were sacrificed after 7 weeks and the kidneys were weighed.

The pro-metastatic activities of SMYD2 and miR-125b were tested in the mouse renal cancer lung metastasis model, as described previously 26. First, the 786-O cells were infected with miR-125b-expression and sponge vectors expressing green fluorescent protein (GFP). Treated cells and control cells (2 × 10^5^) were suspended in 100 μL of PBS and injected intravenously via the tail vein. Each mouse was intraperitoneally injected with AZ505 (40 mg/kg) or the same volume of vehicle-control solution (DMSO) every alternate day. The mice were sacrificed and their lungs were resected at 7 weeks post-injection. The incidences and volumes of metastases were estimated by subjecting the mice to bioluminescence analysis using Living Image software (Xenogen, Baltimore, MD, USA). The photon-emission levels and lung weights were used to assess the relative tumor burden in the mouse lungs.

For the establishment of the subcutaneous xenograft model, BALB/c nude mice were injected subcutaneously with 786-O cells. After 3 weeks, doxorubicin (20 mg/kg), sunitinib (60 mg/kg), and/or AZ505 (40 mg/kg) was injected intraperitoneally into the mice every second day for 4 weeks. The tumor sizes were measured with a pair of vernier calipers and recorded every 4 days; the tumor volumes were calculated using the formula; V = abcπ/6, where a, b, and c represents the length, width, and height of the tumors, respectively. Seven weeks after the subcutaneous injection of the cells, the animals were sacrificed, and their tumors were weighed.

All experiments were approved by the Animal Care and Use Committee of the Tongji Medical College of the Huazhong University of Science and Technology.

### Drug-sensitivity testing and IC_50_ estimation

AZ505-treated and control cells were seeded into 96-well plates. Twelve hours later, the cells were treated with different concentrations of cisplatin, docetaxel, doxorubicin, 5-FU, or sunitinib, or an identical volume of DMSO. Three days after this treatment, the optical density (OD) value of each well was determined using a spectrophotometer. The “relative cell survival” was defined as the number of viable cells in the drug-containing medium/the number of viable cells in the drug-free medium. The “relative cell survival” values and drug concentrations were fitted to dose-response curves to estimate the IC_50_ values of the respective drugs using the SPSS software 27, 28.

### Statistical analysis

Statistical analyses were performed using the SPSS v.22 (IBM, Armonk, NY, USA) and Prism v.6 (GraphPad, La Jolla, CA, USA) software. Comparisons between the SMYD2 expression levels in ccRCC tumor tissues and the paired peritumor tissues were performed using a paired Student's *t* test. Significant associations between SMYD2 expression and clinicopathological parameters were assessed using a χ2 test. Survival curves were plotted using the Kaplan-Meier method and compared with the log-rank test. Cox regression analysis was performed to assess the significance of the variables for survival. Comparisons between two groups were performed using an unpaired Student's *t* test. Differences among the groups were determined by one-way analysis of variance (ANOVA). All the dose-response curve experiments were analyzed by the F-test. The data are presented as the means ± standard deviations (SDs), and P < 0.05 was considered to reflect a statistically significant difference.

## Results

### SMYD2 expression was upregulated in ccRCC and correlated with survival

**Figure 1:**
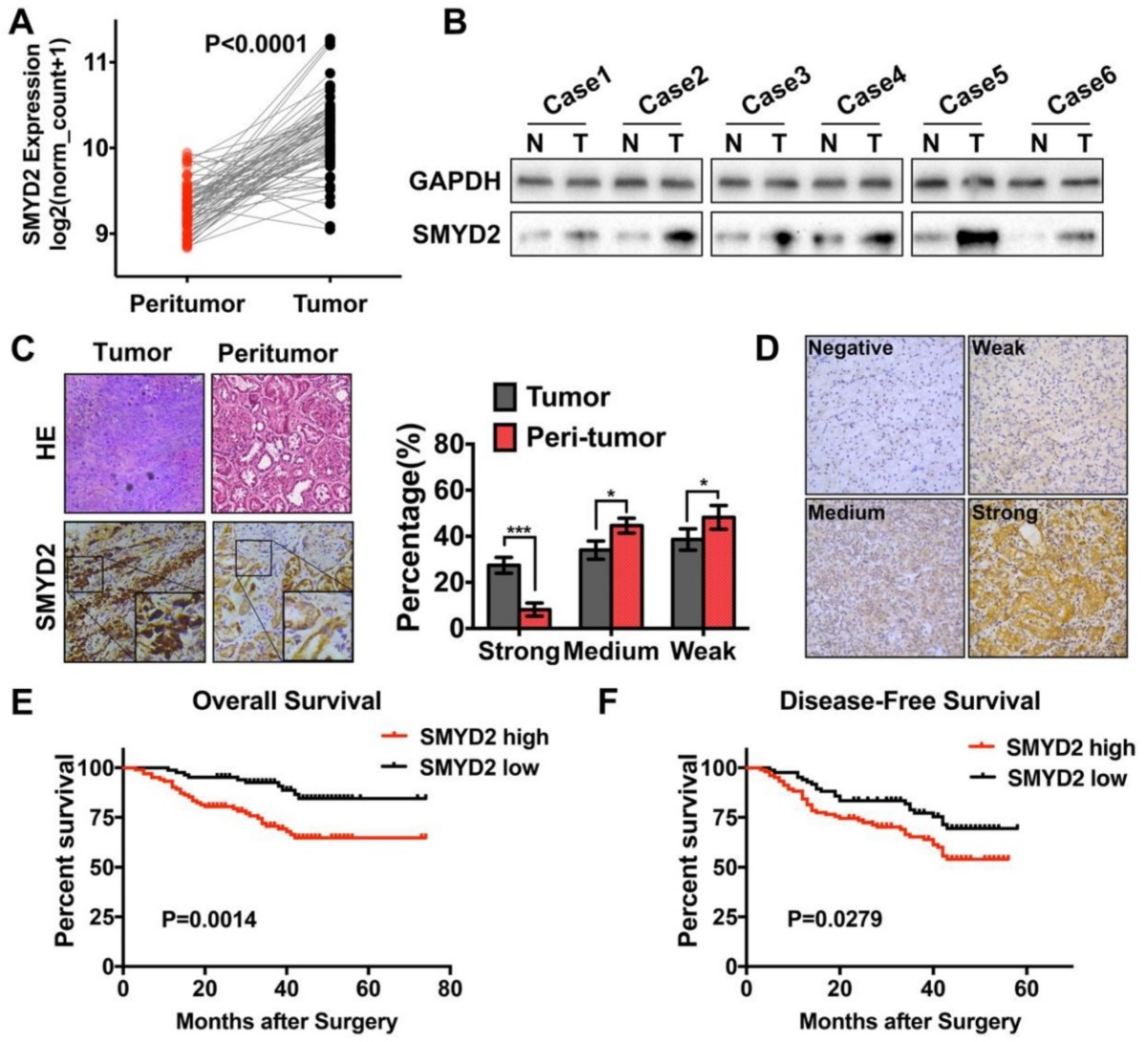
** SMYD2 was upregulated in ccRCC and correlated with worse overall survival and disease-free survival in ccRCC patients. (A)** The expression of SMYD2 in clear cell renal cell carcinoma tissues and their normal counterparts from the TCGA Data Portal (n = 71, paired t test, P < 0.0001). **(B, C)** The expression of SMYD2 in ccRCC tissues and their normal counterparts obtained from 6 cases from the Tongji Hospital, HUST. **(D)** The representative IHC staining intensity of the SMYD2 protein in ccRCC. Negative, weak, medium, and strong SMYD2 expression are shown in order. **(E, F)** Kaplan-Meier overall survival and disease-free survival curves of ccRCC patients according to the expression level of SMYD2 in 186 patients from three medical centers (P = 0.0014 and P = 0.0279, respectively, log-rank).

Processed SMYD2 expression data for the ccRCC samples in the TCGA database were acquired from the UCSC XENA database. The SMYD2 expression levels in the 71 tumor samples were compared to their matched peritumor samples; the results showed that SMYD2 was significantly upregulated in ccRCC (P < 0.0001, Figure 1A). To confirm these findings, we detected the SMYD2 expression in 6 pairs of ccRCC and peritumor specimens from the Tongji Hospital by IHC and western blotting. SMYD2 was widely distributed in the nucleus and cytoplasm of the ccRCC tumor cells. The results showed that the SMYD2 protein was overexpressed in the ccRCC tumor tissues, compared to the case in the paired, adjacent normal tissues (Figure 1B-C).

Expression of the SMYD2 protein in paraffin-embedded ccRCC tissues was determined by IHC, and the clinicopathological significance of SMYD2 in the primary tumor samples was determined based on the IHC-staining pattern of the protein. A total of 186 ccRCC samples were divided into the high SMYD2 expression (n=102) and low SMYD2 expression (n=84) groups, based on the intensity and quantity scores of the SMYD2 staining, as described in the Materials and Methods section. The specific and representative IHC-staining intensity patterns for the SMYD2 protein in the ccRCC samples are shown in Figure 1D. The Kaplan-Meier survival analysis and log-rank test showed that the SMYD2 expression levels in renal cancer cells were significantly associated with a worse overall survival (OS; P = 0.0014) and DFS (P = 0.0279), based on the IRS (Figure 1E and 1F, respectively).

### Correlation between the SMYD2 protein expression levels and clinicopathological characteristics of ccRCC

Associations between the SMYD2 expression levels and clinicopathological parameters are shown in Table S3. High SMYD2 expression was correlated with a higher TNM stage (P = 0.007) and higher incidence of tumor metastasis (P = 0.009). However, SMYD2 was not significantly associated with the patient age, gender, tumor Fuhrman grade, or operation regimen. Notably, high SMYD2 expression was associated with an increased rate of tumor recurrence and disease-related death (P = 0.032 and 0.002, respectively).

The univariate Cox regression analyses demonstrated that the TNM stage, Fuhrman grade, operation regimen, and SMYD2 protein expression were significantly associated with the patients' OS and DFS. The older age of patients contributed to a shorter OS (P = 0.049) but had no significant effect on the DFS (P = 0.105). The multivariate Cox proportional-hazard regression models indicated that high SMYD2 expression (P = 0.022, HR = 2.684) and a higher TNM stage (P = 0.001, HR = 6.710) were independent prognostic factors for poor OS. In addition to SMYD2 expression (P = 0.000, HR = 7.974) and the ccRCC TNM stage (P = 0.000, HR = 7.974), the Fuhrman grade can also serve as an independent predictor of DFS in ccRCC patients (P = 0.023, HR = 2.236, Table 1).

### SMYD2 inhibition suppressed tumor growth *in vitro* and *in vivo*

**Figure 2:**
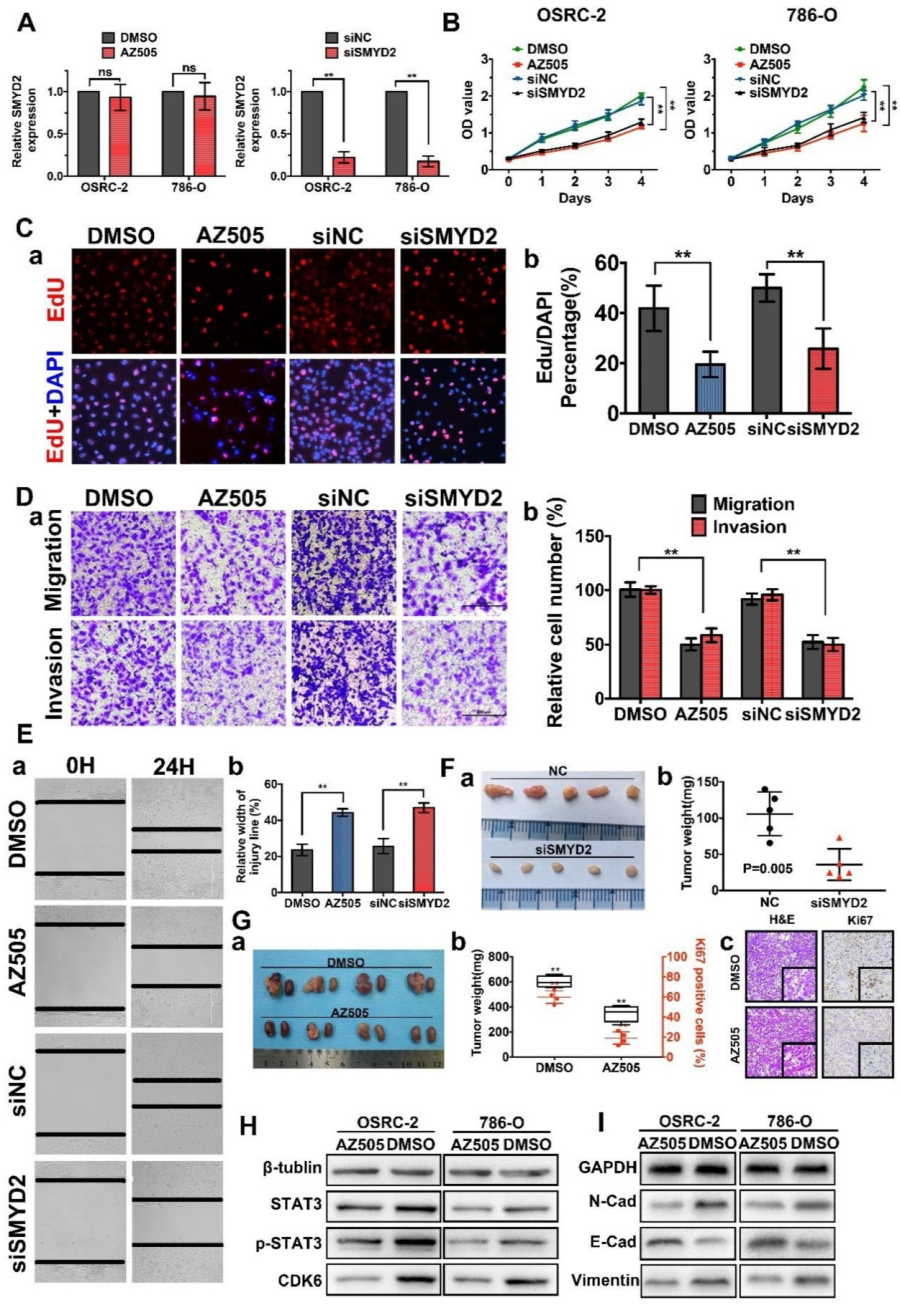
** Inhibition of SMYD2 suppressed cell proliferation, migration, and invasion *in vitro* and *in vivo*. (A)** Relative SMYD2 expression levels after treatment with AZ505 or siSMYD2 are shown. SMYD2-specific siRNA significantly inhibited SMYD2 expression in OSRC-2 and 786-O cells, while AZ505 showed no effect on SMYD2 expression. **(B)** MTS assays were performed to construct the growth curves of the indicated cells. **, P < 0.01. **(C)** Representative micrographs (a) and quantification (b) of EdU-incorporated cells of the indicated cell lines. ** P < 0.01. **(D)** Migration and invasion (Transwell) assays for the indicated renal cancer cells. a, Representative photographs were taken at ×200 magnification. b, The numbers of migrated and invaded cells were quantified in five random images from each group. **, P < 0.01. **(E)** Would-healing assays indicated that AZ505 treatment and SMYD2 knockdown suppressed renal cancer cell migration. Micrographs (a) and the statistic relative width of the injury lines (b) are shown from three independent experiments, **, P < 0.01. **(F)** Subcutaneous xenograft mouse models were established to validate the effects of siSMYD2 *in vivo*. Tumor development was significantly suppressed by siSMYD2, P = 0.005. **(G)** a. The *in vivo* orthotopic xenograft model shows the tumors from the mice in the AZ505-treatment group or DMSO (control)-treatment group; b. Box plot for the average tumor weight (black) and Ki67 expression (red) of tumor samples from the two groups. Tumor weight was evaluated by subtracting the weight of the kidney without injection from the weight of the kidney with injection; c. H&E staining and Ki67 IHC staining of tumors from mice in the two groups. **(H) & (I)** Influence of SMYD2 on the expression of cell proliferation- and EMT-related proteins was tested by western blotting.

To explore and verify the function of SMYD2 as a potential oncogene in renal cancer, we performed cell-viability, cell-proliferation, cell-migration, and cell-invasion assays using cells transfected with SMYD2-specific siRNA (siSMYD2), AZ505-treated cells, and the corresponding negative control cells. AZ505 is an effective and highly selective, competitive inhibitor of SMYD2 that binds in the peptide-binding groove of SMYD2. SMYD2 knockdown in siSMYD2-transfected cells was confirmed by RT-PCR (Figure 2A). The results of the MTS and EdU assays demonstrated that the viability and proliferation of renal cancer cells decreased significantly after the inhibition of SMYD2 via AZ505 treatment or siSMYD2-mediated knockdown (Figure 2B-C). We also found that AZ505 treatment and SMYD2 knockdown inhibited the migration and invasion of 786-O cells, compared to the case for the DMSO or negative control siRNA treated cells, by performing the wound-healing and Transwell assays (Figure 2D-E).

Subcutaneous xenograft mouse models were established to validate the effects of siSMYD2 in vivo, and the results are shown in Figure 2F. Tumor development was significantly suppressed by siSMYD2 (P = 0.005). We further verified the antitumor effects of AZ505 in an orthotopic xenograft model, which was established using BALB/c nude mice. The orthotopic xenograft tumors were resected together with the kidneys from the nude mice 7 weeks after 786-O cell injection, and the tumors were weighed and sliced for pathological observations. Tumor growth in the 786-O cell-implanted nude mice was significantly attenuated by the intraperitoneal injection of AZ505, and the Ki67 expression levels in the tumors from the AZ505-treated mice were significantly suppressed compared to those in the tumors from the mice in the control group (Figure 2G). We also found that the expression levels of cell proliferation and epithelial-mesenchymal transition (EMT)-related proteins were decreased in the AZ505-treated cells, in accordance with results shown above (Figure 2H-I).

### Potential miRNAs correlated with SMYD2 inhibition in RCC development were identified by miRNA microarray profiling

**Figure 3:**
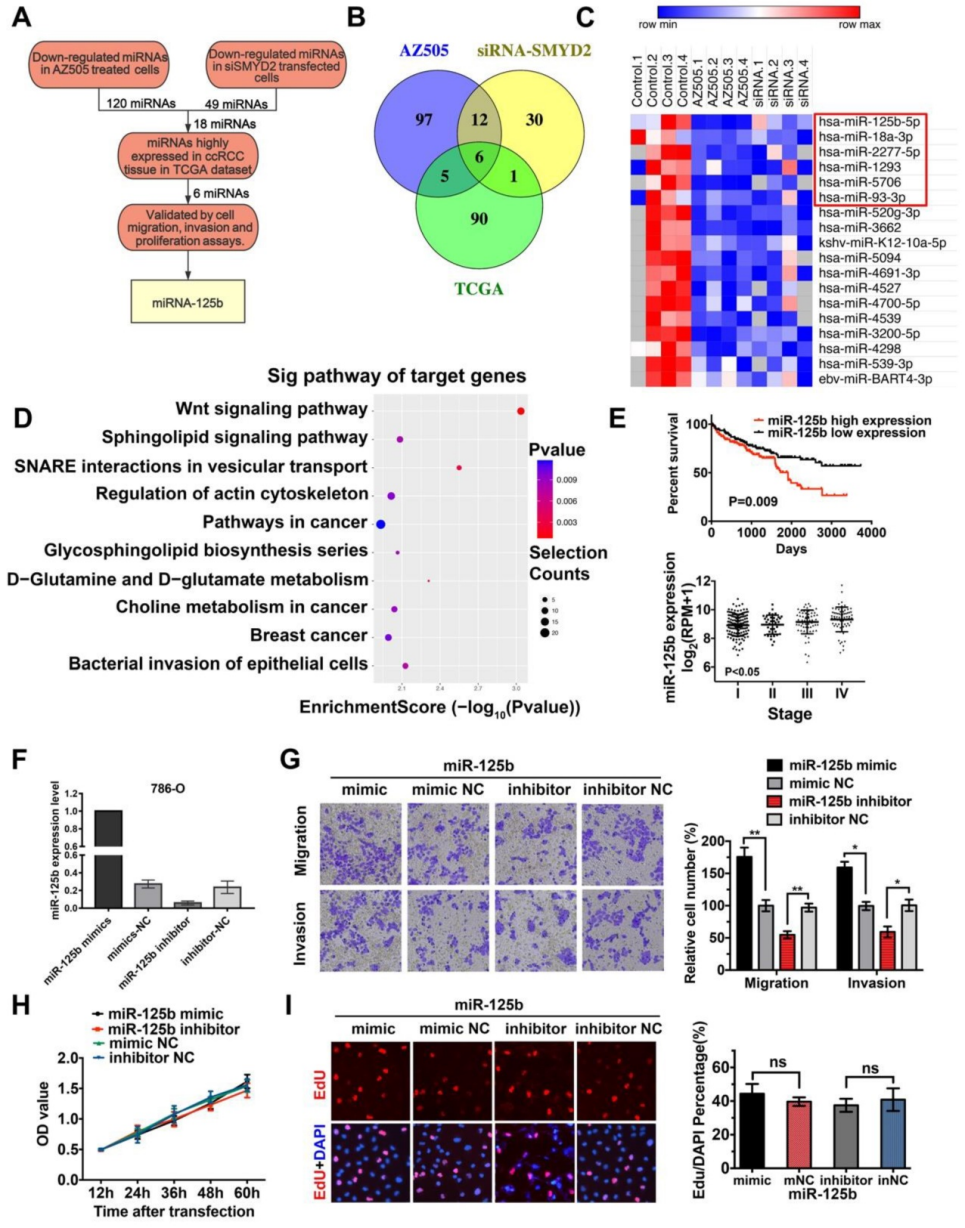
** Identification of miRNAs correlated with SMYD2 inhibition. (A)** The flowchart for screening SMYD2 inhibition-related miRNAs. **(B)** Venn representation of the overlaps among downregulated miRNAs in the AZ505-treated cells, downregulated miRNAs in the siSMYD2-transfected cells, and upregulated miRNAs in the ccRCC samples from the TCGA. **(C)** Heatmap of 18 dysregulated miRNAs in both the AZ505-treated and SMYD2-knockdown cells; the 6 potential downregulated tumor-activating miRNAs are shown in the red box. **(D)** Functional enrichment results of the predicted target genes of the six candidate miRNAs by Gene Ontology and KEGG analysis. **(E)** Kalpan-Meier overall survival curves of ccRCC patients according to the TCGA data for the expression level of miR-125b (P = 0.009, log-rank). The stage of ccRCC patients was correlated with their miR-125b expression (P < 0.05, one-way ANOVA). **(F)** miR-125b expression level in 786-O cells after their transfection with the miR-125b mimics or inhibitors. **(G)** Migration and invasion analyses (Transwell) assays for 786-O cells transfected with the miR-125b mimic or inhibitor and the corresponding control sequence. The number of migrated cells was counted in five random images in case of samples from each treatment group. **(H)** MTS assays for 786-O cells transfected with the miR-125b mimics, miR mimic NC, miR-125b inhibitor, or miR inhibitor NC. **(I)** Representative micrographs and quantification of EdU-incorporated cells from the indicated cell lines. ns, not significant.

It has been reported that the methylation of histone proteins can alter the miRNA expression levels 29. To identify differentially expressed miRNAs, we performed Exiqon miRCURY LNA microarray profiling in AZ505-treated 786-O cells and SMYD2-knockdown 786-O cells for comparison with the cells subjected to the respective control treatment. After quartile normalization, we identified 176 upregulated and 120 downregulated miRNAs in AZ505-treated cells and 3 upregulated and 49 downregulated miRNAs in SMYD2-knockdown cells, with the following criteria: |log_2_ (fold-change)| > 1 and P < 0.05 (Table S4). H3K4 methylation of SMYD2 is an activating modification 30; thus, we focused on miRNAs that were downregulated after SMYD2 suppression (Figure 3A). Eighteen miRNAs were expressed at significantly lower levels in both the AZ505-treated and SMYD2-knockdown cells (Figure 3B-C).

The miRNA expression profiles of 326 kidney clear cell carcinoma samples were downloaded from TCGA, and 150 dysregulated miRNAs (97 upregulated and 53 downregulated) were identified, based on the following cutoff values: |log_2_ (fold-change)| > 1 and P < 0.05 (Figure S2). We screened 6 potentially downregulated tumor-activating miRNAs (miR-18a-3p, miR-2277-5p, miR-1293, miR-93-3p, miR-5706, and miR-125b) by overlapping the 18 downregulated miRNAs in the SMYD2-knockdown cells with the 97 upregulated miRNAs in the ccRCC samples (Fig 3B-C). The miRDB, miRBase, and TargetScan databases were used to predict the potential targets of the six selected miRNAs. The target genes were mapped to GO terms and pathways enriched in the KEGG analysis. The results suggested that the Wnt pathway may play a vital role in the AZ505 treatment-induced inhibition of RCC (Figure 3D and S2).

### MiR-125b functioned as an oncogene by promoting the migration and invasion of ccRCC cells

To validate the tumor growth-promoting effects of the six candidate SMYD2-related miRNAs, and the mimics and inhibitors of the miRNAs were transfected into the renal cancer cell lines. Wound healing, Transwell, MTS, and EdU assays were performed to identify and verify the effects of the miRNAs. The miR-125b expression levels in 786-O cells after their transfection with the miR-125b mimics or inhibitors are shown in Figure 3F. The overexpression of miR-125b promoted the cell mobility, migration, and invasion; however, miR-125b dysregulation did not significantly affect the proliferation of 786-O cells (Figure 3G-I). MiR-18a-3p showed opposing effects, reducing the growth, invasion, and migration of the 786-O cells. No marked differences between the cells transfected with the miRNA mimics and those transfected with the inhibitors of miR-93-3p, miR-1277-5p, miR-5706, and miR-1293 were found in terms of cell proliferation and migration (Figure S3, Table S5). Analysis of TCGA data showed that a high miR-125b expression was significantly associated with a worse survival and high tumor stage in ccRCC patients (Figure 3E).

### SMYD2 regulated the expression of miR-125b, which targets DKK3

**Figure 4:**
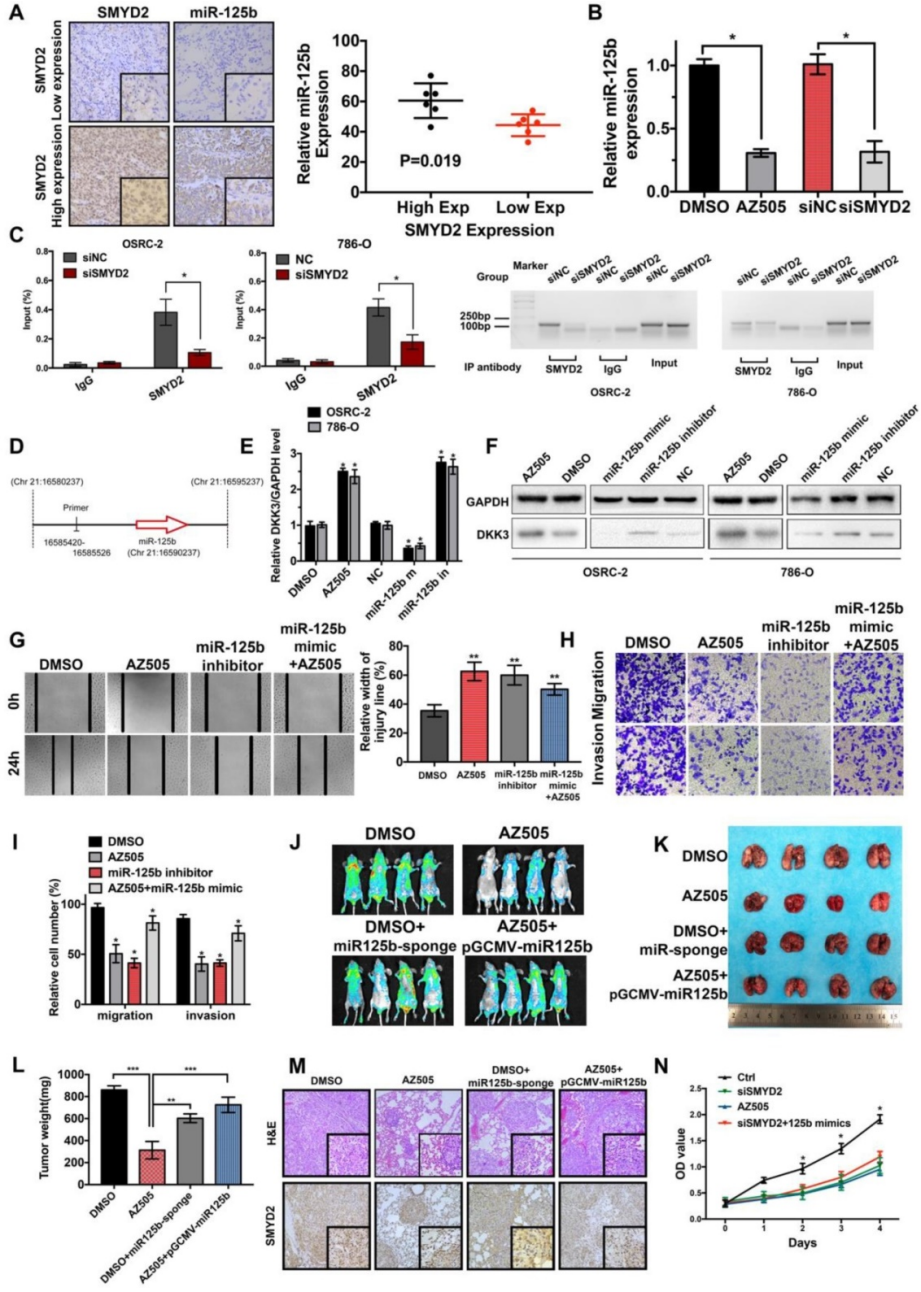
** SMYD2 regulates the expression of miR-125b, which targets DKK3. (A)** The expression of miR-125b in high and low SMYD2 expression ccRCC tissues was determined by *in situ* hybridization, and the results showed that miR-125b was overexpressed in high SMYD2 expression samples (P = 0.019). **(B)** Relative expression of miR-125b in AZ505-, DMSO-, siSMYD2-, and siNC-treated cells using qPCR, indicating that miR-125b expression was reduced in SMYD2-knockdown cells. **(C)** ChIP-qPCR and western blotting analysis showing the enrichment of SMYD2 at a region lying upstream of miR-125b in 786-O and OSRC-2 cells. *, P < 0.05. **(D)** Schematic showing the genomic location of miR-125b. The upstream regions used for ChIP-PCR are indicated using black lines. **(E)** qPCR analysis of the DKK3 expression in renal cancer cell lines treated with AZ505 and DMSO, or transfected with the miR-125b mimics, miR-125b inhibitor, and negative control siRNA (n=3). Student's t-test, *, P < 0.05. The error bars in the panels indicate the SDs. **(F)** Western blot analysis of DKK3 and GAPDH expression in OSRC-2 and 786-O cells treated with AZ505 and DMSO, or transfected with the miR-125b mimics, miR-125b inhibitor, or NC siRNA. **(G), (H), and (I)** Rescue experiment of SMYD2 and miR-125b *in vitro*. The results of the wound healing assays (G) and Transwell assays (H and I) suggested that AZ505 and the miR-125b inhibitor inhibited the migration and invasion of renal cancer cells, and the miR-125b mimic rescued the inhibition induced by AZ505. **(J), (K), (L), and (M)** Rescue experiment of SMYD2 and miR-125b in the mouse 786-O lung metastasis model. The *in vivo* renal cancer lung metastatic bioluminescent model showed that miR-125b mimic rescued the tumor growth suppression induced by the inhibition of SMYD2. (J) Representative bioluminescent images of the lungs of nude mice at the 50th day after the IV injection of the renal cancer cells. (K) Photographs of the lungs excised 7 weeks after the injection of the 786-O cells through the caudal vein of the nude mice. (L) Lung weight of each nude mouse at the end of 7 weeks. ** P < 0.01. (M) H&E staining and SMYD2 IHC staining of the lung metastatic tumors. (N) MTS assays verified that miR-125b did not reverse the SMYD2 knockdown-induced suppression of the proliferation of renal cancer cells.

The miR-125b expression levels in the six tumor samples each with the highest and lowest SMYD2 expression levels were detected by ISH (Figure S1); the results indicated that the miR-125b expression was elevated in the samples from the high SMYD2 expression group, compared to those in the low SMYD2 expression group (Figure 4A). The qPCR results also showed that the miR-125b expression decreased after AZ505 treatment or siSMYD2-mediated SMYD2 knockdown (Figure 4B). The ChIP assays showed that SMYD2 occupied a genomic upstream region of miR-125b and regulated the miR-125b expression (Figure 4C-D). The target gene-prediction database and Lu et al. showed that miR-125b bound directly to the 3′-untranslated region of DKK3, which has been identified to serve as an important regulatory factor in the WNT pathway and act as a tumor suppressor in renal cancer 31. In addition, the qPCR and western blotting analyses confirmed the effects of the miR-125b mimics and inhibitors on the regulation of DKK3 expression, and showed that SMYD2 suppression downregulated the DKK3 expression at both the mRNA and protein levels (Figure 4E-F).

### Effects of SMYD2 on tumor progression were reversed by miR-125b

Based on our findings, we evaluated whether SMYD2 knockdown could reduce miR-125b expression, which would be consistent with the observation of DKK3 overexpression. Thus, we tested whether the exogenous manipulation of miR-125b affected the function of SMYD2. The wound-healing and Transwell assays showed that the transfection with the miR-125b inhibitors blocked the migration and invasion of 786-O cells and that the transfection with the miR-125b mimics reversed the suppression of proliferation and migration caused by AZ505 treatment (Figure 4G-I). Moreover, we found that miR-125b knockdown inhibited the tumor growth *in vivo* and that miR-125b overexpression partially reversed the inhibitory effects of AZ505 on the colonization of lung metastatic tumors in the tail vein xenograft model (Figure 4J-M). Collectively, these results suggested that the effects of SMYD2 on cell migration and invasion were mediated, at least in part, through miR-125b. Meanwhile, we performed MTS assays to verify whether miR-125b could reverse the SMYD2 knockdown-mediated suppression of the proliferation of renal cancer cells; the results showed that miR-125b could not reverse this suppression (Figure 4N).

### Inhibition of SMYD2 synergized with anticancer drugs in renal cancer

**Figure 5:**
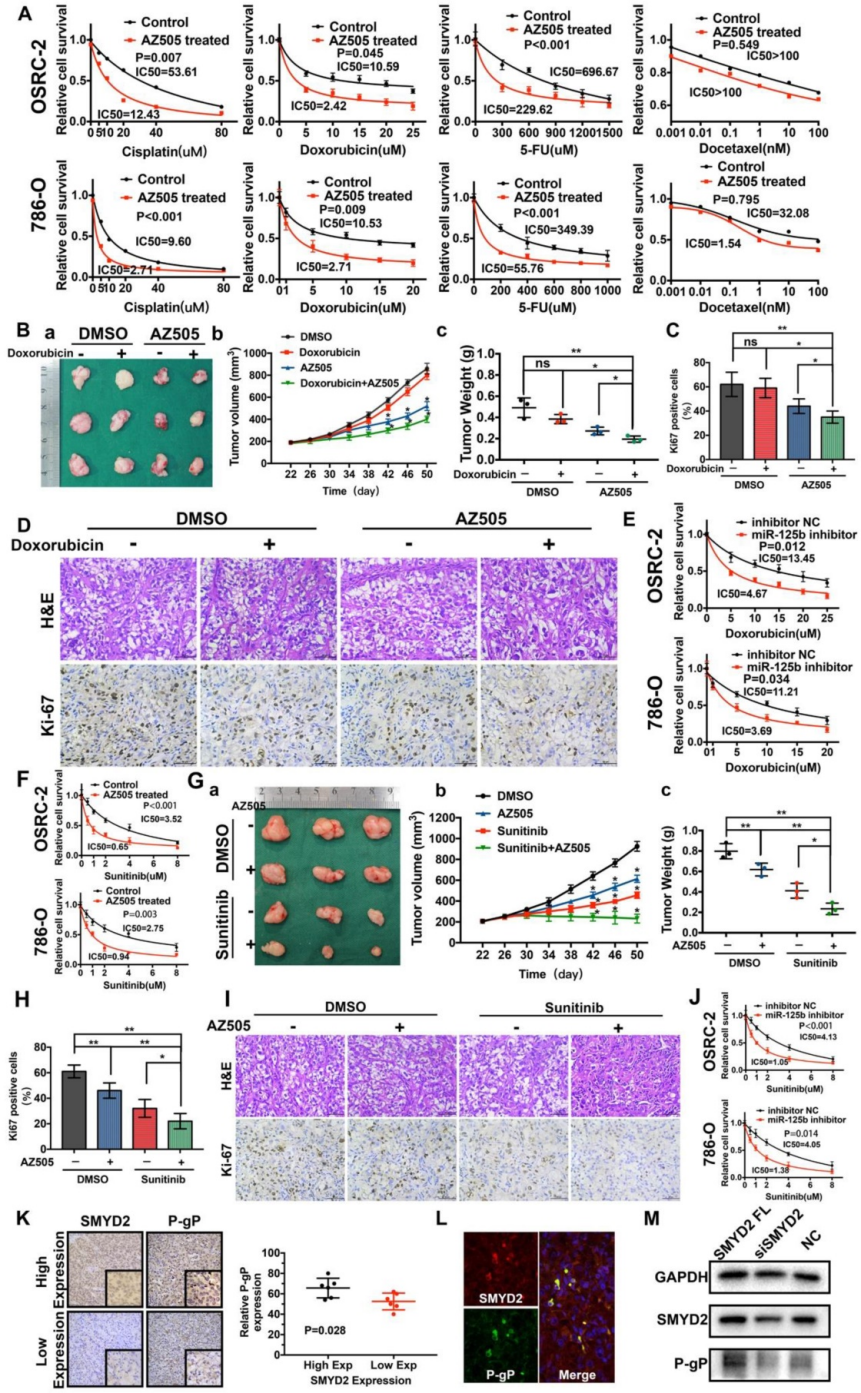
** The SMYD2 inhibitor and miR-125b inhibitor synergized with anti-tumor drugs to show therapeutic effects against renal cancer *in vitro* and *in vivo* by regulating P-gP. (A)** AZ505 enhanced the sensitivity of the renal cancer cell lines OSRC-2 and 786-O to multiple anticancer drugs. The IC_50_ for each treatment is presented. **(B)** Combination therapy with doxorubicin and AZ505 in a renal cancer subcutaneous xenograft model. (a) Representative images of the tumors isolated from the mice in each treatment group. (b, c) Tumor volumes and weights were quantified (*P < 0.05, **P < 0.01, ns: not significant). **(C, D)** Subcutaneous xenograft tumors were characterized by Hematoxylin and eosin (H&E) staining, and the proliferation of the tumor cells was characterized by Ki67 staining (*P < 0.05, **P < 0.01, ns: not significant). **(E)** The miR-125b inhibitor enhanced the response to doxorubicin in the renal cancer cell lines. **(F)** Enhanced response to sunitinib following AZ505 treatment is shown in the OSRC-2 and 786-O cell lines. **(G)** Combined treatment with AZ505 and sunitinib in a kidney cancer mouse model. (a) Photographs of tumors excised from the nude mice subjected the indicated treatments. (b, c) Tumor volumes and weights were quantified (*P < 0.05, **P < 0.01). **(H, I)** Hematoxylin and eosin (H&E) staining and Ki67 IHC staining of tumors from the mice in the different groups. **(J)** The miR-125b inhibitor enhanced the sensitivity of renal cancer cells to sunitinib. The IC_50_ for each cell line is presented. **(K)** IHC staining for P-gP in ccRCC tissues with high and low SMYD2 expression (P = 0.028). **(L)** Immunofluorescence of SMYD2 (red), P-gP (green), and nuclei (blue). **(M)** Western blot analysis of SMYD2, P-gP, and GAPDH expression in SMYD2-overexpressing, AZ505-treated, or DMSO (control)-treated 786-O cells.

Pioneering studies have shown that SMYD2 is associated with drug resistance in non-small-cell lung cancer and esophageal cancer 20, 21. The IC_50_ values of four different chemotherapy drugs for AZ505-treated cells and control cells were estimated. The estimated IC_50_ values of cisplatin, doxorubicin, or 5-FU (but not docetaxel) for AZ505-treated RCC cells were significantly lower than those for the control cells, indicating that the SMYD2 inhibition enhanced the drug sensitivity in renal cancer cells (Figure 5A). Next, a subcutaneous xenograft model was used to evaluate the antitumor efficacy of the co-treatment with AZ505 and doxorubicin. In the mice from the monotherapy group, which were treated with doxorubicin alone, no significant antitumor efficacy was observed, compared to the case in those treated with the vehicle control (DMSO). On the other hand, AZ505 treatment resulted in the decrease of the tumor sizes and weights in the mice. Following the combination treatment with AZ505 and doxorubicin, the tumor volumes and weights were significantly reduced (Figure 5B), and the number of Ki67-positive cells was decreased (Figure 5C-D). Notably, the miR-125b inhibitor enhanced the sensitivity of the renal cancer cells to doxorubicin (Figure 5E).

Similarly, the synergistic effects of AZ505 and sunitinib were confirmed *in vitro* and *in vivo*. The IC_50_ of sunitinib for AZ505-treated cells was significantly lower than that for the control cells (Figure 5F). AZ505 and sunitinib treatment alone in the tumor-bearing nude mice suppressed the growth of renal tumors (Figure 5G). Moreover, a significant reduction in renal cancer growth was observed following the combination treatment with AZ505 and sunitinib, compared to the case for the samples from the control and monotherapy groups, as demonstrated by the decreased number of Ki67-positive cells (Figure 5H-I). Therefore, combining an SMYD2 inhibitor with a chemotherapeutic agent resulted in synergistic therapeutic effects against renal cancer *in vivo*. Moreover, the miR-125b inhibitor improved the response of renal cancer cells to sunitinib, demonstrating that miR-125b is a master miRNA that affects the sensitivity of cancer cells to multiple anticancer drugs (Figure 5J).

### SMYD2 inhibition drove P-gP (MDR-1) down-regulation in RCC

The P-gP expression levels in six ccRCC specimens each with the highest and lowest SMYD2 expression levels were measured by IHC (Figure S1); the results indicated that P-gP was upregulated in the tumor tissues with high SMYD2 expression (Figure 5K). Double immunofluorescent staining revealed the co-localization of the SMYD2 and P-gP proteins in renal cancer tissues (Figure 5L). In addition, the P-gP expression increased in SMYD2-overexpressing cells and decreased after the transfection of the cells with the SMYD2 siRNA (Figure 5M).

## Discussion

SMYD2 is a putative oncogene that regulates its downstream target genes via histone or non-histone methylation and affects the proliferation, apoptosis, metastasis, and chemosensitivity of tumor cells 12, 32. Besides ccRCC, abnormal expression and tumorigenic properties of SMYD2 have recently been reported in many tumors, including papillary thyroid carcinoma, esophageal squamous cell carcinoma, gastric cancer, hepatocellular carcinoma and papillomavirus-unrelated non-multiple head and neck carcinomas 16-19, 33. The research most relevant to kidney disease has shown that SMYD2 promotes cyst growth in autosomal-dominant polycystic kidney disease 34. In this study, SMYD2 was found to be upregulated, and patients with higher SMYD2 expression showed a poor prognosis among our cohort of 186 ccRCC cases diagnosed across three medical centers in China; our results also showed that SMYD2 appears to play an oncogenic role in RCC, particularly in ccRCC.

In recent years, the interest in molecular targeting using competitive SMYD2 inhibitors for treating various diseases has increased 21, 34-36. AZ505 was chosen as an SMYD2 inhibitor because its effects were more efficient compared to those of other SMYD2 inhibitors 37. Combined with efficient SMYD2 inhibition and the use of siSMYD2, miRNAs regulated by SMYD2 were detected via the miRNA microarray analysis, and six downregulated miRNAs were selected for further studies. Functional assays and ChIP validation revealed miR-125b as a downstream target of SMYD2. Interestingly, miR-125b has been found to play inconsistent roles in different tumors. For example, miR-125b has been found to promote tumor progression in cervical cancer, breast cancer, lung adenocarcinoma, and gastric cancer, but attenuate malignancy in hepatocellular carcinoma 38-44. Our results showed that the miR-125b-induced promotion of cell proliferation, invasion, and metastasis could be reversed by AZ505. Thus, miR-125b acted as a critical downstream target of SMYD2 and played a key role in the oncogenicity of ccRCC.

Notably, accumulating evidence has shown that SMYD2 is associated with the drug resistance of human cancers, including non-small cell lung cancer and esophageal cell carcinoma 20, 21. Previous findings have also confirmed that the dysregulation of miRNAs, including miR-125b, plays important roles in the protective effects of chemotherapy against cancer. MiR-125b can regulate the chemo-resistance of several types of cancer cells. For example, Yu et al. have found that miR-125b overexpression triggered the activation of the CXCL12/CXCR4 axis during the EMT and invasion of colorectal cancer cells, conferring 5-FU resistance to these cells by dysregulating autophagy 45. Abdi et al. have revealed that miRNAs such as miR-125b (an onco-miRNA) play key roles in regulating drug resistance and p53 tumor-suppressor activity in multiple myeloma 46. Considering that SMYD2 regulates p53 activity, our results demonstrate the importance of the SMYD2/miRNA-EMT/MDR pathway in drug resistance and the development of ccRCC. Our study also revealed that SMYD2 inhibition led to the down-regulation of P-gP, which is an ATP-binding cassette multidrug transporter 47, thus enhancing the chemotherapeutic drug sensitivity in RCC cells.

However, a previous study by Pires-Luís *et.al* has revealed that low SMYD2 expression is associated with worse disease-specific survival and disease-free survival 48, which is contradictory to the findings of our study. Pires-Luís *et.al* collected 160 renal cell tumor samples, consisting of four different histological subtypes, including ccRCC, pRCC, chRCC and renal oncocytoma. However, our study only focused on ccRCC, the most common subtype of RCC, and analyzed a cohort of 186 ccRCC cases diagnosed across three medical centers in China. The heterogeneity of the tumor histological subtypes of tumors in their study may have led to the contradictory conclusion. Moreover, the patients in our study were enrolled from medical centers in China, and their baseline, the treatment they received, and postoperative intervention were not the same as those in European countries.

In summary, our findings demonstrate that SMYD2 was overexpressed and acted as a tumor promotor in ccRCC. It was an independent biomarker for the poor prognosis of ccRCC patients. Inhibition of SMYD2 suppressed the progression of ccRCC *in vivo* and *in vitro* by dysregulating miR-125b, which acted as a tumor promoting factor in ccRCC. Additionally, SMYD2 and miR-125b expression may be essential for the development of MDR in ccRCC cells. SMYD2/miR-125b-targeted therapies may be considered a novel strategy for reversing the resistance of cancer cells to chemotherapy treatment. Thus, SMYD2, which may serve as a new molecular diagnostic marker and therapeutic target for treating ccRCC, is of great significance and may be suitable for several therapeutic applications in clinical settings.

## Supplementary Material

Supplementary figures and tables 2, 3, 5.

Supplementary table 1.

Supplementary table 4.

## Figures and Tables

**Table 1:** Cox regression analysis of prognostic factors on overall survival and disease-free survival

	Overall survival						Disease free survival					
	Univariate			Multivariate			Univariate			Multivariate		
Variables	HR	P-value	95% CI	HR	P-value	95% CI	HR	P-value	95% CI	HR	P-value	95% CI
Age (>58yr vs ≤58yr)	1.873	**0.049**	1.004-3.493	1.073	0.857	0.498-2.314	1.505	0.105	0.918-2.468	0.915	0.799	0.468-1.810
Gender (Male vs Female)	1.008	0.984	0.466-2.178	1.692	0.271	0.663-4.317	1.359	0.373	0.692-2.669	1.536	0.295	0.688-3.429
TNM stage (Stage III,IV vs Stage I,II)	12.038	**0.001**	4.278-33.874	7.148	**0.001**	2.339-21.847	6.945	**0.001**	3.523-13.689	8.716	**0.001**	2.980-25.498
Fuhrman Grade (G3-4 vs G1-2)	2.154	**0.045**	1.015-4.568	1.585	0.250	0.724-3.470	2.864	**0.002**	1.478-5.549	2.206	**0.026**	1.101-4.421
Operation (Radical vs Partial)	6.016	**0.013**	1.453-24.903	/	/	/	2.957	**0.012**	1.275-6.858	/	/	/
SMYD2 expression (High vs Low)	3.001	**0.002**	1.475-6.107	2.646	**0.024**	1.138-6.154	1.763	**0.031**	1.052-2.954	2.027	**0.048**	1.006-4.084
