# Inhibition of SMYD2 suppresses tumor progression by down-regulating microRNA-125b and attenuates multi-drug resistance in renal cell carcinoma: Erratum

**DOI:** 10.7150/thno.68810

**Published:** 2022-01-01

**Authors:** Libin Yan, Beichen Ding, Haoran Liu, Yangjun Zhang, Jin Zeng, Junhui Hu, Weimin Yao, Gan Yu, Ruihua An, Zhiqiang Chen, Zhangqun Ye, Jinchun Xing, Kefeng Xiao, Lily Wu, Hua Xu

**Affiliations:** 1 Department of Urology, Tongji Hospital, Tongji Medical College, Huazhong University of Science and Technology, Wuhan, China;; 2 Institute of Urology of Hubei Province, Wuhan, China; 3 Department of Urinary Surgery, First Affiliated Hospital of Harbin Medical University, Harbin, Heilongjiang, China.; 4 Department of Urology, The First Affiliated Hospital of Xiamen University, Xiamen, China.; 5 Department of Urology, The People's Hospital of Shenzhen City, Shenzhen, China.; 6 Department of Molecular and Medical Pharmacology, David Geffen School of Medicine, University of California at Los Angeles, Los Angeles, USA

The authors regret that the original version of our paper unfortunately contained some incorrect representative images. The EdU images of AZ505 groups in Figure 2Ca and the invasion image of siSMYD2 in Figure 2Da have been misused during figure assembly. The correct version of the Figure 2 appears below.

The authors confirm that the corrections made in this erratum do not affect the original conclusions. The authors apologize for any inconvenience or misunderstanding that the errors may have caused.

## Figures and Tables

**Figure 2:**
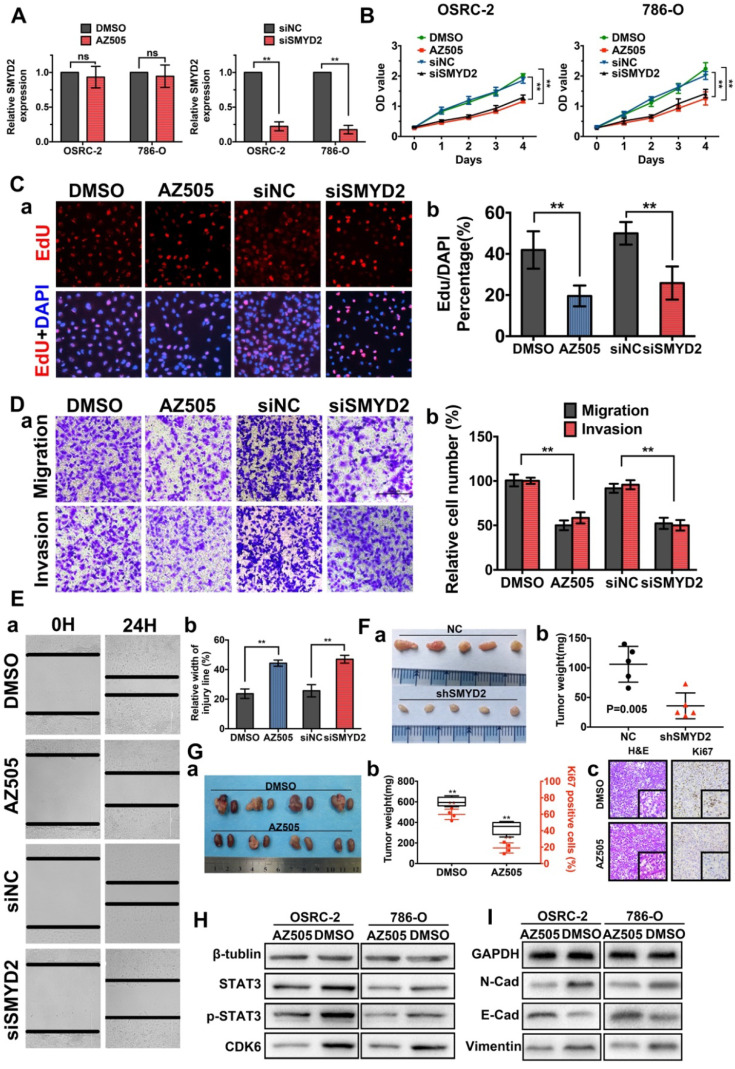
**Inhibition of SMYD2 suppressed cell proliferation, migration, and invasion *in vitro* and *in vivo*.** (A) Relative SMYD2 expression levels after treatment with AZ505 or siSMYD2 are shown. SMYD2-specific siRNA significantly inhibited SMYD2 expression in OSRC-2 and 786-O cells, while AZ505 showed no effect on SMYD2 expression. (B) MTS assays were performed to construct the growth curves of the indicated cells. **, P < 0.01. (C) Representative micrographs (a) and quantification (b) of EdU-incorporated cells of the indicated cell lines. ** P < 0.01. (D) Migration and invasion (Transwell) assays for the indicated renal cancer cells. a, Representative photographs were taken at ×200 magnification. b, The numbers of migrated and invaded cells were quantified in five random images from each group. **, P < 0.01. (E) Would-healing assays indicated that AZ505 treatment and SMYD2 knockdown suppressed renal cancer cell migration. Micrographs (a) and the statistic relative width of the injury lines (b) are shown from three independent experiments, **, P < 0.01. (F) Subcutaneous xenograft mouse models were established to validate the effects of siSMYD2 *in vivo*. Tumor development was significantly suppressed by siSMYD2, P = 0.005. (G) a. The *in vivo* orthotopic xenograft model shows the tumors from the mice in the AZ505-treatment group or DMSO (control)-treatment group; b. Box plot for the average tumor weight (black) and Ki67 expression (red) of tumor samples from the two groups. Tumor weight was evaluated by subtracting the weight of the kidney without injection from the weight of the kidney with injection; c. H&E staining and Ki67 IHC staining of tumors from mice in the two groups. (H) & (I) Influence of SMYD2 on the expression of cell proliferation- and EMT-related proteins was tested by western blotting.

