# Supplementary material for: Inhibition of SMYD2 suppresses tumor progression by down-regulating microRNA-125b and attenuates multi-drug resistance in renal cell carcinoma
Source: Theranostics. 2019 Oct 22;9(26):8377–91. doi: 10.7150/thno.37628 (PMC6857066; doi:10.7150/thno.37628)
Supplement: Supplementary file 1 — Supplementary figures and tables 2, 3, 5. [file thnov09p8377s1.pdf]

Inhibition of SMYD2 suppresses tumor progression by down-regulating microRNA-125b and attenuates multi-drug resistance in renal cell carcinoma

Libin Yan[1,2#], Beichen Ding[1,3#], Haoran Liu[1,2], Yangjun Zhang[1,2], Jin Zeng[1,2], Junhui Hu[1,2], Weimin Yao[1,2], Gan Yu[1,2], Ruihua An[3], Zhiqiang Chen[1,2], Zhangqun Ye[1,2] , Jinchun Xing[4], Kefeng Xiao[5], Lily Wu[6], Hua Xu[1, 2*]

1, Department of Urology, Tongji Hospital, Tongji Medical College, Huazhong University of Science and Technology, Wuhan, China;
2, Institute of Urology of Hubei Province, Wuhan, China
3, Department of Urinary Surgery, First Affiliated Hospital of Harbin Medical University, Harbin, Heilongjiang, China.
4, Department of Urology, The First Affiliated Hospital of Xiamen University, Xiamen, China.
5, Department of Urology, The People's Hospital of Shenzhen City, Shenzhen, China.
6, Department of Molecular and Medical Pharmacology, David Geffen School of Medicine, University of California at Los Angeles, Los Angeles, USA

[#]These authors have contributed equally to this work.

Figure S1

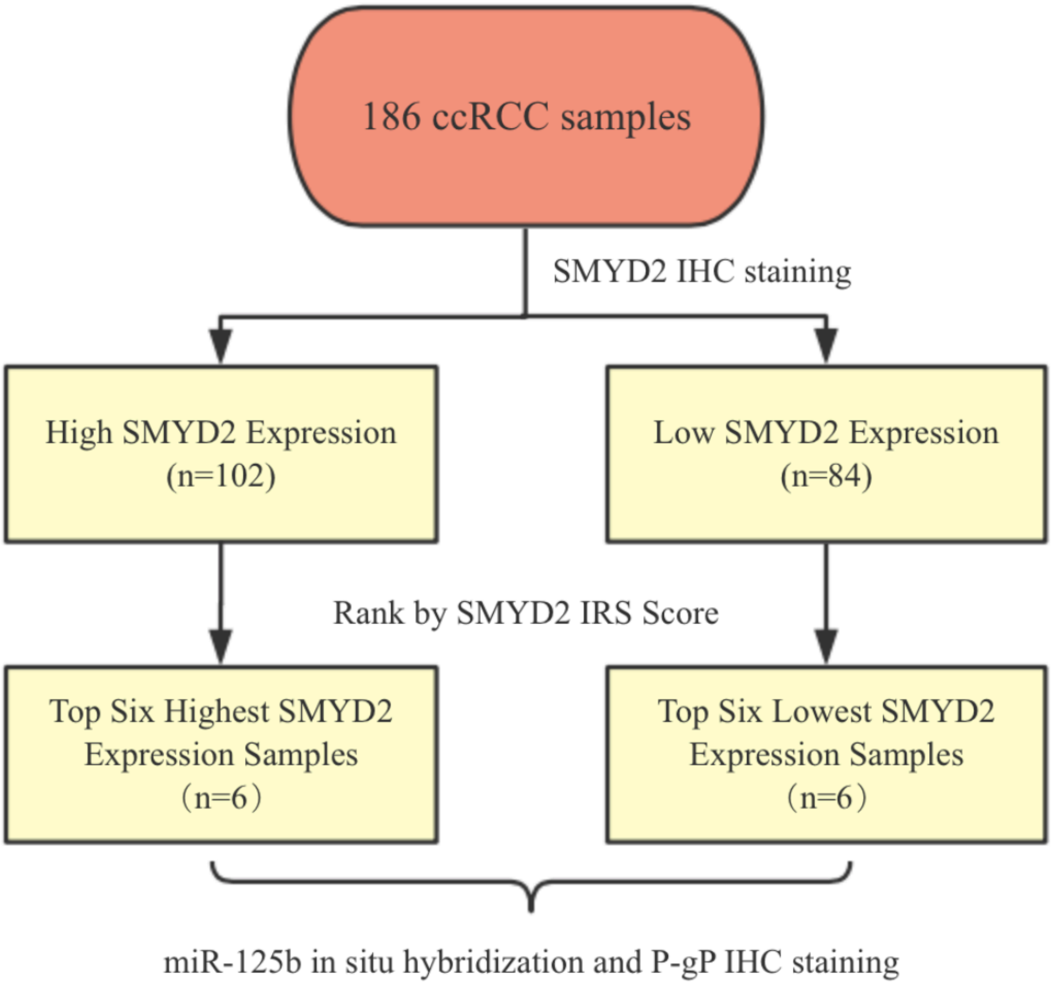

Figure S1 The flowchart was drawn to expound the criterion of sample selection for miR-125b in situ hybridization and P-gP IHC staining.

Figure S2

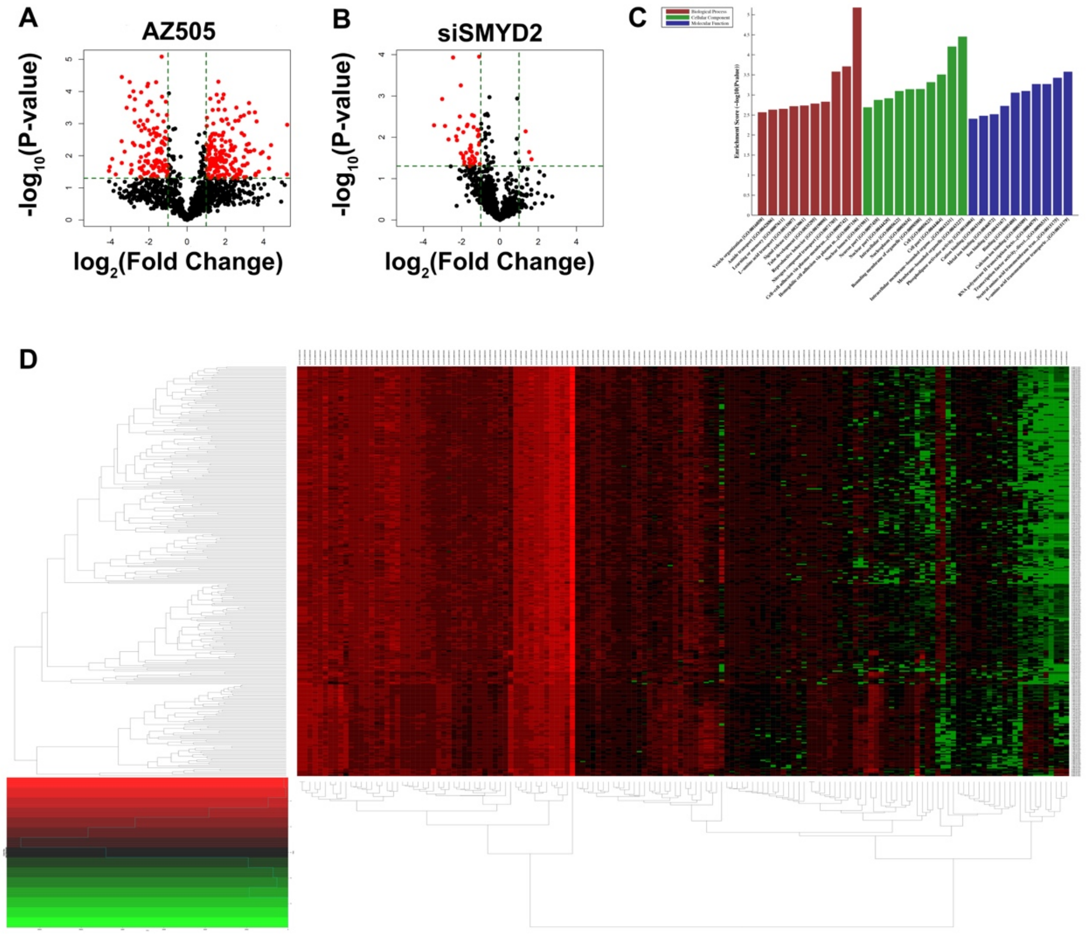

Figure S2. **Process of screening miRNAs.**

(A) and (B) Volcano map describes the differentially expressed miRNAs after AZ505 and siSMYD2 interference.

(C) Gene Ontology Enrichment analysis of target genes of the 6 selected miRNAs.

(D) Differentially expressed miRNAs in ccRCC from TCGA database. $|log_2(Foldchange)|>1$ and $P<0.05$.

Figure S3

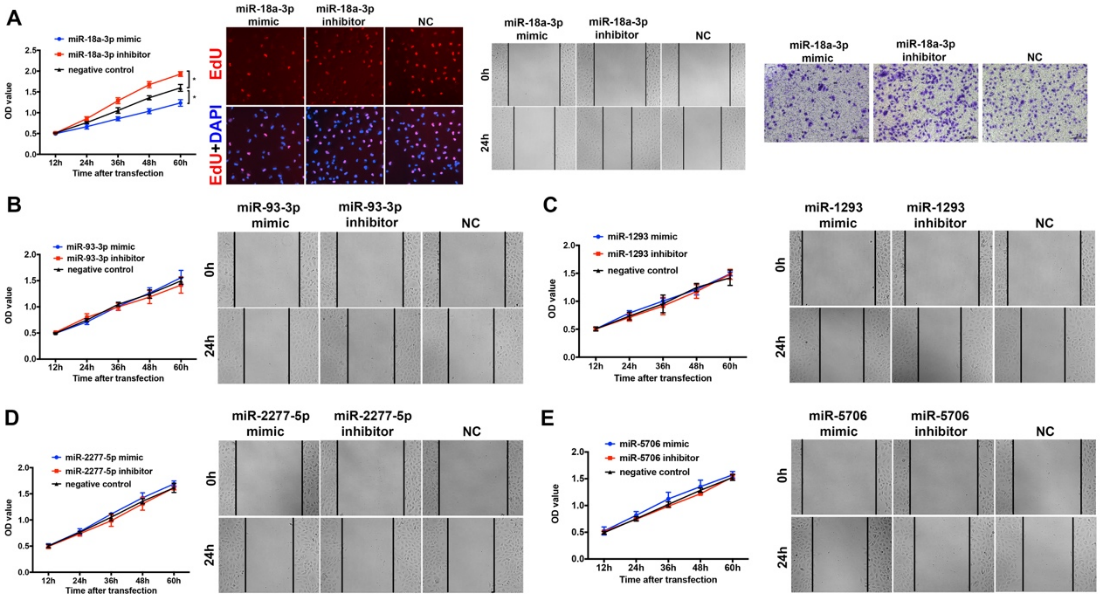

Figure S2. **Verifying the effects of candidate miRNAs on RCC cells.**

(A) miR-18a-3p inhibited the growth, invasion and migration of renal cancer cells.

(B),(C),(D) and (E) miR-93-3p, miR-1277-5p, miR-5706 and miR-1293 showed no obvious effect on cell proliferation and migration.

Table.S2 Sequences of siRNA and miRNA oligonucleotides

Name	Sequence (5'-3')
siSMYD2	Sense GAUUUGAUUCAGAGUGACA
	Antisense UGUCACUCUGAAUCAAAUC
hsa-miR-1293 mimic	UGGGUGGUCUGGAGAUUUGUGC
hsa-miR-1293 inhibitor	GCACAAAUCUCCAGACCACCCA
hsa-miR-2277-5p mimic	AGCGCGGGCUGAGCGCUGCCAGUC
hsa-miR-2277-5p inhibitor	GACUGGCAGCGCUCAGCCCGCGCU
hsa-miR-5706 mimic	UUCUGGAUAACAUGCUGAAGCU
hsa-miR-5706 inhibitor	AGCUUCAGCAUGUUAUCCAGAA
hsa-miR-93-3p mimic	ACUGCUGAGCUAGCACUUCCCG
hsa-miR-93-3p inhibitor	CGGGAAGUGCUAGCUCAGCAGU
hsa-miR-18a-3p mimic	ACUGCCCUAAGUGCUCCUUCUGG
hsa-miR-18a-3p inhibitor	CCAGAAGGAGCACUUAGGGCAGU
hsa-miR-125b mimic	UCCCUGAGACCCUAACUUGUGA
hsa-miR-125b inhibitor	UCACAAGUUAGGGUCUCAGGGA

Table.S3 Associations between clinicopathological parameters and SMYD2 expression in ccRCC patients

	n	SMYD2 expression		P-value
		Low	High	
Total	186	84	102	
Age (year)				0.669
58 or younger	95(51.1%)	42	53	
Older than 58	91(48.9%)	42	49	
Gender				0.399
Male	149 (80.1%)	70	79	
Female	37 (19.9%)	14	23	
TNM stage				**0.007**
I-II	86(46.2%)	48	38	
III-IV	100(53.8%)	36	64	
Fuhrman grade				0.840
G1-2	107 (57.5%)	49	58	
G3-4	79 (42.5%)	35	44	
Metastasis at Diagnosis				**0.009***
No	178(95.7%)	84	94	
Yes	8(4.3%)	0	8	
Operation				0.759
Radical	148 (79.5%)	66	82	
Partial	38 (20.5%)	18	20	
Status of patients				**0.002**
Alive	144(77.4%)	74	70	
Disease related-death	42(22.6%)	10	32	
Recurrence				**0.032**
No evidence	122(65.6%)	62	60	

Yes	64(34.4%)	22	42	

TNM, tumor node metastasis; Radical, radical nephrectomy; Partial, partial nephrectomy

P-values are from chi-square or Fisher's exact test and were statistically significant at P<0.05, statistically significant values are in bold; * Fisher's exact test

Table.S5 The effects of 6 candidate miRNAs on RCC cells.

miRNA	MTS assay	Edu assay	Wound healing assay	Transwell Invasion
miR-125b	No effect	No effect	+	+
miR-18a-3p	-	-	-	-
miR-93a-3p	No effect	/	No effect	/
miR-1293	No effect	/	No effect	/
miR-2277-5p	No effect	/	No effect	/
miR-5706	No effect	/	No effect	/

+， tumor promoting； -， tumor suppressing； /, no data.
